# Genome-Wide Identification and Characterization of the *RCI2* Gene Family in Allotetraploid *Brassica napus* Compared with Its Diploid Progenitors

**DOI:** 10.3390/ijms23020614

**Published:** 2022-01-06

**Authors:** Weiqi Sun, Mengdi Li, Jianbo Wang

**Affiliations:** 1 State Key Laboratory of Hybrid Rice, College of Life Sciences, Wuhan University, Wuhan 430072, China; weiqisun@whu.ed.cn (W.S.); mengdili@whu.edu.cn (M.L.); 2 Key Laboratory of Resource Biology and Biotechnology in Western China, Ministry of Education, College of Life Sciences, Northwest University, Xi’an 710069, China

**Keywords:** polyploidization, allotetraploid, *Brassica napus*, *RCI2* gene family, phylogenetic analysis, *cis*-element

## Abstract

*Brassica napus* and its diploid progenitors (*B. rapa* and *B. oleracea*) are suitable for studying the problems associated with polyploidization. As an important anti-stress protein, RCI2 proteins widely exist in various tissues of plants, and are crucial to plant growth, development, and stress response. In this study, the *RCI2* gene family was comprehensively identified and analyzed, and 9, 9, and 24 *RCI2* genes were identified in *B. rapa*, *B. oleracea*, and *B. napus*, respectively. Phylogenetic analysis showed that all of the identified *RCI2* genes were divided into two groups, and further divided into three subgroups. Ka/Ks analysis showed that most of the identified *RCI2* genes underwent a purifying selection after the duplication events. Moreover, gene structure analysis showed that the structure of *RCI2* genes is largely conserved during polyploidization. The promoters of the *RCI2* genes in *B. napus* contained more *cis*-acting elements, which were mainly involved in plant development and growth, plant hormone response, and stress responses. Thus, *B. napus* might have potential advantages in some biological aspects. In addition, the changes of *RCI2* genes during polyploidization were also discussed from the aspects of gene number, gene structure, gene relative location, and gene expression, which can provide reference for future polyploidization analysis.

## 1. Introduction

The formation and evolution of polyploid species play an important role in the evolution of most plants [1,2]. In recent years, researchers have concluded that nearly all angiosperms have experienced at least one polyploidy (genome doubling) in their evolutionary history [3,4,5]. Polyploids are divided into autopolyploids and allopolyploids [6]. Allopolyploidization refers to the hybridization and doubling of different sets of chromosomes from two or more species [7,8]. In addition to the effects of genome multiplication, there is also the effect of genome heterozygosity because genomes are non-homologous [9,10]. The impact will eventually lead to changes in plant genomes [11]. At the early stage of polyploid synthesis, the continuous instability of chromosomes often leads to the occurrence of unequal chromosome separation and chromosome loss [12,13]. In addition to changes in the DNA level, the epigenetic level and gene expression level also change during polyploidization [14,15]. The correlation between DNA methylation in rice genomes and the evolution of repeated genes during ancient polyploidization events has also been demonstrated in rice methyltransferase mutants [14], and transposon amplification was found in allotetraploid tobacco [15]. Gene expression changes in allopolyploidization are a common phenomenon, and usually include parental gene silencing, activation, and some homologous gene expression changes [16,17,18]. In the study of plant polyploid, the loss or amplification of genes, structural changes, and expression patterns are highly valued by scholars.

RCI2 (Rare-cold-inducible) proteins, which are often named as plasma membrane proteins 3 (PMP3) or low temperature induced proteins 6 (Lti6), are present as multigene family encoding proteins in plants. In general, the RCI2 protein locates in the plasma membrane, endoplasmic reticulum, or Golgi apparatus [19]. The *RCI2* gene responds to various stresses, especially low temperature, and it may affect seed germination and root growth [20,21]. The absence of PMP3 can lead to the hyperpolarization of membrane potential, the excessive accumulation of Na^+^, and even more sensitivity to salt damage [22,23,24]. On the contrary, plant *RCI2* gene expression can depolarize membrane potential by reducing cations uptake and anions excretion [25]. Ultimately, salt tolerance is improved. Studies have shown that *CsRCI2D* contributes positively to seed germination and the post-germination seedling growth of *Camelina* *sativa* under high salinity stress [19]. A freezing injury may cause cell dehydration, and AtRCI2A B can stabilize membrane proteins [24,26]. In abiotic stress, the tolerance of the *RCI2* gene mutant, wild-type, and overexpressed plants increased in turn [20,21]. The *RCI2* gene family is involved in a wide range of complex life activities in plants, and plays an important role in plant resistance to the adversity and regulation of growth and development [23,27]. Therefore, it is necessary to comprehensively analyze the structural characteristics, phylogeny, and molecular evolution of the *RCI2* gene family to reveal the breeding role of the *RCI2* gene family in *B. napus* and its diploid ancestors.

*Brassica* is not only a big genus in Brassicaceae, but also one of the most economically valuable genera. According to the chromosome number and homology of *Brassica*, Nagaharu (1935) summarized the interspecific relationships of six *Brassica* species in the form of U’s Triangle, including three diploid basic species and three tetraploid compound species [28]. The three basic species *B. rapa* (AA, 2n = 2x = 20), *B. nigra* (BB, 2n = 2x = 16), and *B. oleracea* (CC, 2n = 2x = 18) crossed with each other and formed *B. juncea* (AABB, 2n = 4x = 36), *B. napus* (AACC, 2n = 4x = 38) and *B. carinata* (BBCC, 2n = 4x = 34) by chromosome doubling. At present, the identification and analysis of the *RCI2* gene family have been carried out in many plants, for example: *Arabidopsis thaliana* [29], *Zea mays* [25], *Cucumis sativus* L. [30], *Triticum aestivum* [31], and the function of individual members of the family has been explored [32,33,34]. However, the effect of whole-genome polyploidization and hybridization on the *RCI2* gene family in *Brassica* is not clear. In this study, the *RCI2* gene family in *B. napus* and its diploid progenitors were studied in detail based on genomic data, and compared the *RCI2* gene family of allotetraploid with its diploid ancestors, which will help us to better understand the mechanism of genetic variation after polyploidization, and provide help for the further molecular breeding of *B. napus* and other polyploid varieties.

## 2. Results

### 2.1. Identification and Phylogenetic Analysis of RCI2 Gene Family Members

The RCI2 proteins of *B. napus* and its diploid progenitors, which had the same conserved domain with Arabidopsis RCI2 proteins, were obtained by BLSATp in BRAD database, and then the corresponding genes encoding RCI2 proteins were found. To identify the *RCI2* genes in *B. napus* and its diploid progenitors, eight AtRCI2 protein sequences were obtained, and the BLASTp program was used to query the BRAD database. The results showed that 10, 7, and 23 genes were selected as candidate genes in *B. rapa*, *B. oleracea*, and *B. napus*, respectively. Then, by entering the gene ID of the *RCI2* gene in the Arabidopsis genome, we searched the BRAD database for syntenic genes, which complemented the first method. Through this method, we added 1, 4, and 2 members in *B. rapa*, *B. oleracea*, and *B. napus*, respectively. Then, three public protein databases (Pfam, SMART, and CDD) were used to search for the PMP3 domain in the protein sequences encoded by the candidate *RCI2* genes, and the proteins that did not contain the complete conserved PMP3 domain were removed. Finally, 9, 9, and 24 genes were identified as *RCI2* genes in *B. rapa*, *B. oleracea*, and *B. napus*, respectively. Table S1 shows that the *RCI2* gene family information in *B. napus* and its diploid ancestors.

We named *Brassica* *RCI2* gene family members according to the homologous relationship with *RCI2* genes in *A. thaliana*. There are nine *RCI2* genes in *B. rapa* (*BrRCI2A-a* to *BrRCI2H-b)*, nine *RCI2* genes in *B. oleracea* (*BoRCI2A-a* to *BoRCI2H-b)*, and twenty-four *RCI2* genes in *B. napus* (*BnARCI2A-a* to *BnARCI2H-b2)*. The letters “A–H” in the name correspond to eight *RCI2* genes in Arabidopsis, and the last lowercase letter indicates the degree of homology with corresponding genes in Arabidopsis. “a” represents the highest homology and “e” represents the lowest homology. If several genes have the same homology, the numbers 1, 2, and 3 are added to distinguish them. In addition, we also analyzed the physical and chemical characteristics of 42 RCI2 proteins, the molecular weights (MW), theoretical PI values, instability index (II), grand average of hydropathicity (GRAVY), and aliphatic index (Table S2). The results showed that the length of all RCI2 proteins in the three species varied from 50 to 170 aa, and the molecular weights varied from 5000 to 20,000 KDa. The instability index of 19 RCI2 proteins were greater than 40, and 40 RCI2 proteins were acidic proteins (pI < 7).

**Figure 1:**
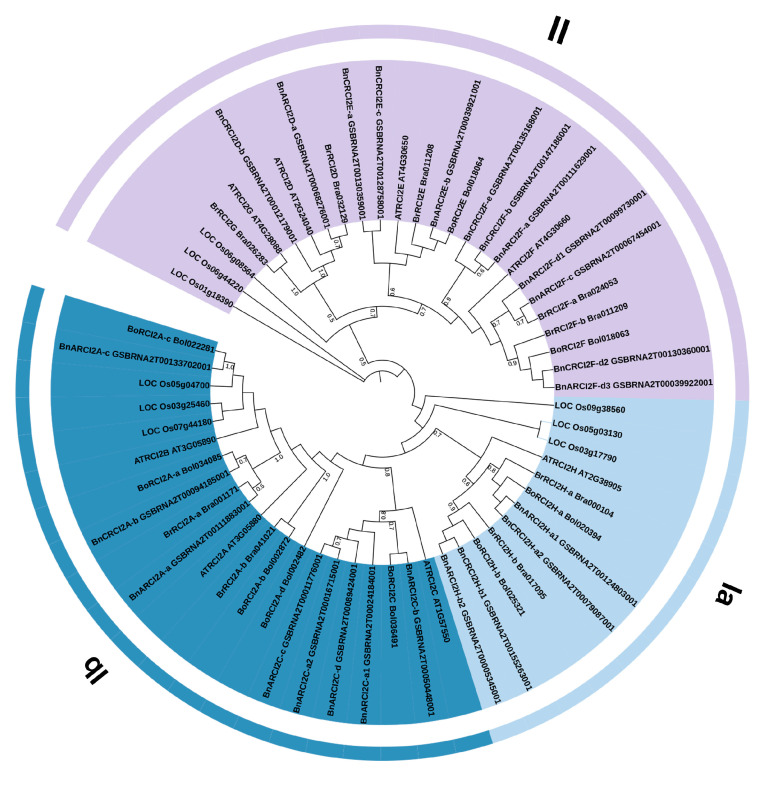
Phylogenetic tree of *RCI2* gene family in *B. rapa*, *B. oleracea*, and *B. napus*, Arabidopsis and *Oryza sativa*, including 59 RCI2 proteins, was divided into two groups (I and II) and further divided into three subgroups (Ia, Ib and II). A bootstrap value greater than 50% was displayed at the base of the branch.

In order to analyze the evolution and phylogeny of *RCI2* genes, a phylogenetic tree was constructed from the proteins encoded by *RCI2* genes. RCI2 protein sequences from five different species were used as reference sequences to construct a phylogenetic tree, including *A. thaliana* (8), *Oryza sativa* (9), *B. rapa* (9), *B. oleracea* (9), and *B. napus* (24). According to the length of the proteins encoded by *RCI2* genes and whether the C-terminal is hydrophilic, we can get three subgroups (Ia, Ib, and II) [35,36]. There were 12 genes on branch Ia, 21 genes on branch Ib, and 26 genes on branch II (Figure 1). Generally, the higher the bootstrap values at the base of each branch in the phylogenetic tree, the higher the reliability relationship of genes clustered at the end of the branches. When bootstrap values are ≥50%, the bootstrap values are displayed in the phylogenetic tree. By comparing the distance of the branches of the *RCI2* genes between the three *Brassica* species with *A. thaliana* and *O. sativa*, the homology of *RCI2* genes between three species with the *A. thaliana* was higher than *O. sativa*.

### 2.2. Chromosomal Localization of the RCI2 Genes

**Figure 2:**
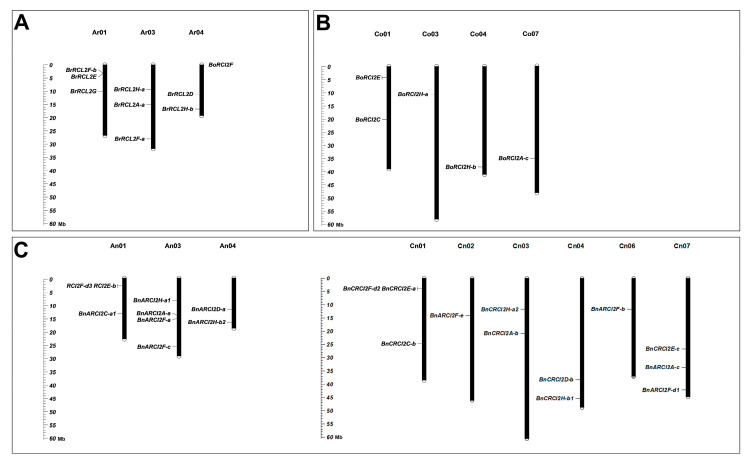
The chromosomal location of the *RCI2* gene family in *B. rapa* (**A**), *B. oleracea* (**B**) and *B. napus* (**C**).

According to the physical locations of all *RCI2* gene family members in *B. rapa*, *B. oleracea*, and *B. napus* genomes searched in BRAD database, the distribution of *RCI2* genes on chromosomes was analyzed. Eight of the nine *BrRCI2* genes were located on three chromosomes, six of the nine *BoRCI2* genes on four chromosomes, nine of the eleven *BnARCI2* genes on three chromosomes, and twelve of the thirteen *BnCRCI2* genes on six chromosomes (Figure 2). By comparing gene localization on the chromosomes of *B. rapa* A_r_ and *B. napus* A_n_, the genes on the corresponding chromosomes are not only homologous genes, but also have the same relative position. The comparison result of *B. oleracea* C_o_ and *B. napus* C_n_ chromosomes is the same. It can be found that A_n_03 has more *BnARCI2F-C* gene than A_r_03, which indicates that gene amplification occurs on the same chromosome in the process of allopolyploidization. Comparing *B. oleracea* C_o_ with *B. napus* C_n_ chromosomes, we found that gene amplification occurred not only on the same chromosome, but also between different chromosomes.

### 2.3. Synteny and Duplicated Gene Analysis

**Figure 3:**
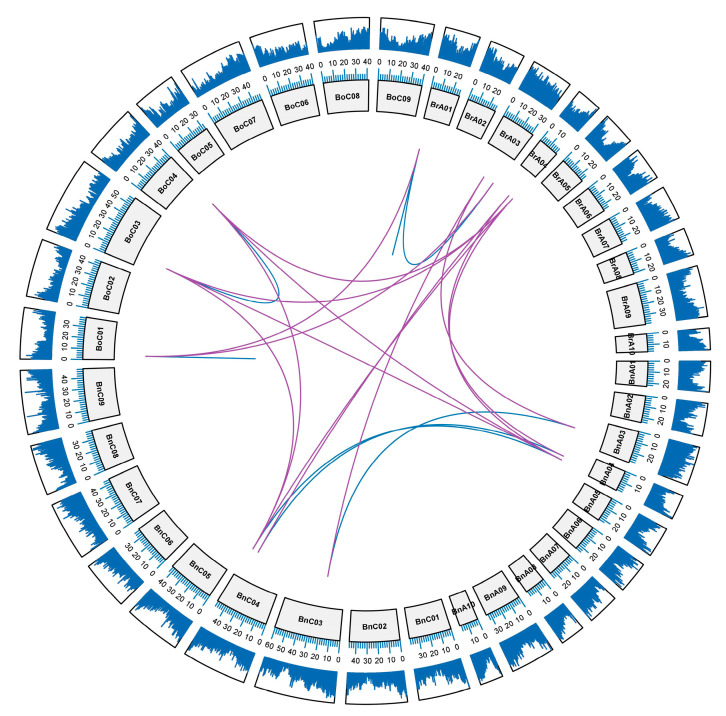
Genome-wide synteny analysis of A_n_ and C_n_ subgenome in *B. napus*, and A_r_ genome in *B. rapa*, and C_o_ genome in *B. oleracea*. Blue lines in the figure represented the paralogous genes and purple represented orthologous genes. The outer ring was gene density on chromosome. The inner circle was the chromosome name and its length scale.

An analysis of the synteny of the *RCI2* gene in *B. napus* and its diploid progenitors (*B. rapa* and *B. oleracea*) genome will help to better understand the amplification pattern of genes during *B. napus* formation. Synteny mainly describes the connections between genome segments of different species derived from the same ancestor, which is of great significance for revealing the genomic evolution of a related species [37]. We searched syntenic *RCI2* genes in BRAD database and analyzed syntenic *RCI2* genes in *A. thaliana*, *B. rapa*, *B. oleracea*, and *B. napus*. There were 8 *RCI2* syntenic genes in *B. rapa*, 9 *RCI2* syntenic genes in *B. oleracea*, and 6 *RCI2* syntenic genes in *B. napus* searched in the BRAD database. A total of 23 *RCI2* genes were searched in the syntenic region, accounting for 55% of the identified *RCI2* genes, indicating that a large number of *RCI2* genes in *B. napus* and its diploid ancestors were lost in the evolutionary process. Through the BRAD database, we screened a total of 26 pairs of genes, and made a Circos graph of these gene pairs (Figure 3). It indicated that 26 pairs of *RCI2* genes were divided into two parts, among which gene pairs within the same specie were called paralogs (5 pairs of genes, connected by blue lines) and those between different species were called orthologs (21 pairs of genes, connected by purple lines). We also looked at the location of the 26 pairs of genes on chromosomes and the distribution of gene density on each chromosome. There were two blue lines that look as though they were truncated; in actuality, they were two pairs of tandem duplication genes (*BrRCI2E*-*BrRCI2F-b*, *BoRCI2E*-*BoRCI2F*).

To verify whether the *RCI2* gene family of *B. napus* and its diploid progenitors are affected by selection pressure during evolution, we used a BLASTn and syntenic gene search in the BRAD database to identify duplicated gene pairs among them. There were 36 identified segmental duplication gene pairs, among which 1, 2, and 33 gene pairs were found in *B. rapa*, *B. oleracea*, and *B. napus*, respectively. We calculated the Ka, Ks, and Ka/Ks values of duplication gene pairs. These values, evolutionary patterns, and time were then showed in Table S3. Three of the 36 duplicated gene pairs had no Ka/Ks value, because they have the same genetic sequence. As for the remaining 33 pairs, except for the *BnARCI2C-a2* and *BnCRCI2C-c*, the Ka/Ks values of all gene pairs were less than 1, which proved that the most duplication gene pairs were affected by the purifying selection.

### 2.4. The Gene Duplication Types of RCI2 Genes

The gene duplication types can be classified as whole-genome duplication (WGD), Tandem duplication (TD), proximal duplication (PD), transposed duplication (TRD), and dispersed duplication (DSD) [38]. As shown in Table 1, a large number of WGD were present in the *RCI2* gene families of *B. napus* and its diploid progenitors. The WGD proportions of *B. rapa*, *B. oleracea*, and *B. napus* were 100%, 56%, and 54%, respectively. The single-gene replication of *RCI2* genes were observed, mainly involving TD, TRD, DSD, and PD. The mechanism of DSD has not been clearly studied, so it was excluded in the following analysis. We found that PD, TD, and TRD accounted for the highest proportion in *B. rapa* (100%), and TD had the highest proportion in *B. oleracea* (67%), while TRD had the most duplication types in *B. napus* (58%). We found that TD was very common in the two diploids, which was similar to the previous study on the *RCI2* gene family in *A. thaliana*. However, the occurrence frequency of TRD in *B. napus* was the highest, and higher than that of WGD. Therefore, we speculate that the TRD mediated by transposon is critical for the expansion of the *RCI2* gene family in *B. napus* during the allopolyploidization process.

### 2.5. Structural Analysis of RCI2 Genes

**Figure 4:**
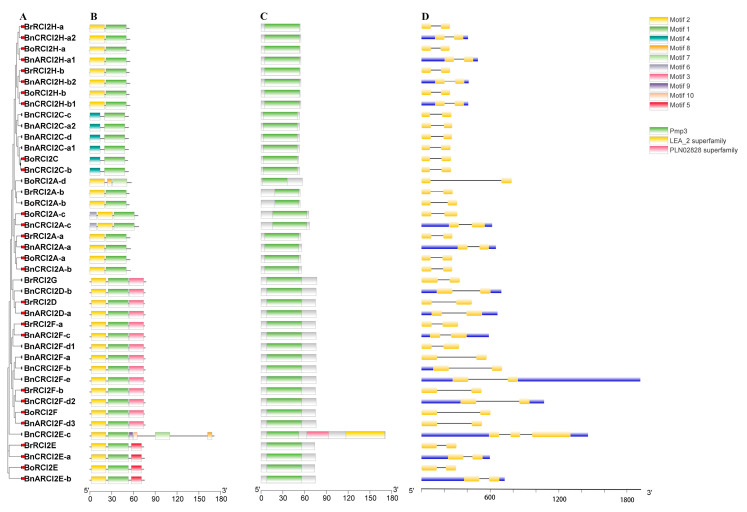
Intron/exon structure and conserved domain and motif overlap characterizations of the *RCI2* gene family in *B. rapa* and *B. oleracea*, *B. napus*. The characterizations include phylogenetic tree of the *RCI2* gene family (**A**), domain location (**B**), and conserved motif location (**C**), intron/exon structure (**D**). Note: Orthologous gene pairs (14 pairs) between *B. napus* and its diploid ancestors may have direct evolutionary relationships, marked by the red circle.

The exon-intron structure of each *RCI2* gene was obtained by comparing the cDNA sequence with its own gene sequence (Figure 4). As can be seen from Figure 4, the intron-exon structure in *RCI2* gene family was very conservative. Except for *BnCRCI2E-c*, which had three exons and two introns, the rest were all two exons and one intron. The motif was analyzed by MEME online software, and a total of 10 motifs were identified. The number and distribution pattern also had been shown (Figure 4). There are 41 RCI2 proteins containing motif 1 and 36 RCI2 proteins containing motif 2, which indicates that motif 1 and motif 2 are conserved in the *RCI2* gene family in *B. rapa*, *B. oleracea*, and *B. napus*. We also studied the conserved domains of the RCI2 proteins of three species. As can be seen from Figure 4, all proteins except for the *BnCRCI2E-c* protein only contain the PMP3 domain. To further study the changes of gene structure during allopolyploidization, we selected 14 homologous pairs that may have a direct evolutionary relationship between *B. napus* and two ancestral species, and found that the internal exon-intron structure and motif distribution was the same for each pair. Therefore, the *RCI2* genes were very conserved during allopolyploidization.

### 2.6. Sequence Alignment of All Identified RCI2 Proteins

**Figure 5:**
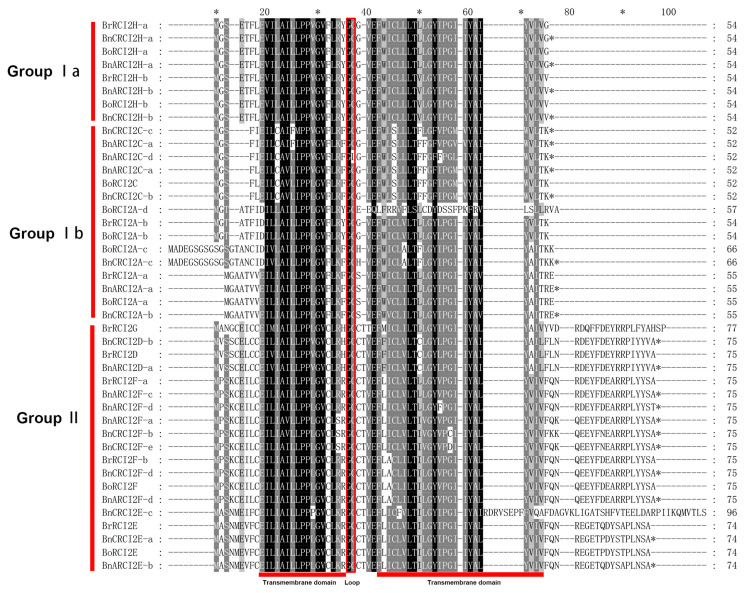
Sequence alignment of all identified *RCI2* genes. Multiple sequence alignment was conducted using ClustalX and visualized in GeneDoc. The asterisk (*) at the top of the protein sequence indicates that the amino acid at this position is very conserved. While the asterisk (*) at the end of the protein sequence indicates that there is a termination codon.

To study the homology domain sequence characteristics of the RCI2 protein, multiple sequences were compared based on the RCI2 protein sequence of *B. napus* and its diploid progenitors, and similar or identical residues were covered with different shades of color. As shown in Figure 5, all RCI2 proteins of the three species consisted of two hydrophobic transmembrane structures and a loop that separates them. These RCI2 proteins were divided into two groups. The RCI2 proteins in Group I had a short C-terminal, and the RCI2 proteins in Group II had a hydrophilic charged residue of about 20 amino acids at the C-terminal. Group Ia RCI2s possess hydrophobic C-terminal ends while Group Ib RCI2s contain hydrophilic C-terminal ends [35,36]. An additional conserved Cys residue was found between the two conserved hydrophobic domains of most Group II RCI2 proteins.

### 2.7. Subcellular Localization Analysis of RCI2 Proteins

Cell-PLoc online software was used to predict the subcellular localization of 42 RCI2 proteins (Table S2), and the results showed that most of the RCI2 proteins were localized in cell membrane (26). In addition, the remaining RCI2 proteins localized in vacuoles (15) and chloroplast (1). Through the analysis of the subcellular localization of 14 pairs of orthologous genes (Figure 4), it was shown that all BnRCI2 proteins were located in the same organelle as the corresponding homolog of RCI2 in its diploid progenitors. It indicated that the *RCI2* gene family was highly conserved in subcellular localization during the allopolyploidization.

### 2.8. Analysis of Cis-Acting Elements in the Promoters of RCI2 Genes

**Figure 6:**
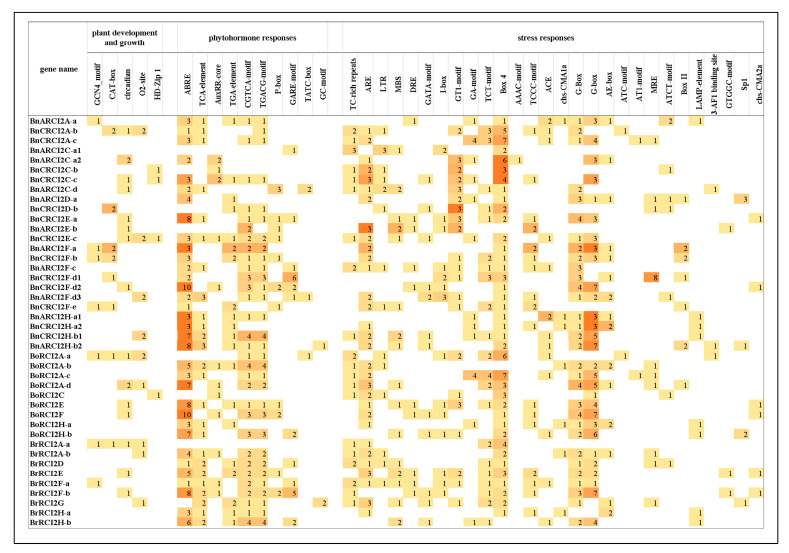
*Cis*-acting elements on promoters of all identified *RCI2* genes. From the picture, there is a positive correlation between the value of the number and the shade of the color in each grid.

The PlantCARE database was used to predict the *cis*-acting elements upstream 2000 bp of *RCI2* gene family in *B. napus* and its diploid ancestors. We identified *cis*-acting elements in all *RCI2* gene promoters responsible for plant development and growth (5), plant hormone response (10), and stress responses (29) in *B. napus* and its two diploid progenitors (Figure 6). Plant hormone response elements and stress response elements are crucial for plant defense regulation. In *B. napus*, plant development elements varied from 1 to 8, plant hormone response elements varied from 1 to 6, and stress responses elements varied from 1 to 7. As for *RCI2* genes in *B. rapa*, plant hormone response elements and stress responses elements varied from 1 to 8 and 1 to 7. However, all the number of plant development and growth elements which were contained by *BrRCI2* was 1. The number ranges of *cis*-acting elements in *B. oleracea* are as follows: the plant development and growth elements varied from 1 to 2, the plant hormone response elements varied from 1 to 10, and the number of stress responses elements varied from 1 to 7. All *RCI2* genes in *B.* *oleracea* did not contain CAG-motif and GTGGC-motif. Compared with *cis*-acting elements in *B. napus* and *B. oleracea*, the number and types of *cis*-acting elements in *B. rapa* were the least. Overall, ABRE, Box4, and G-box were significantly more present than other elements in *B. napus* and its diploid ancestors, which indicated that these three kinds of *cis*-acting elements were more conservative in *Brassica* transcription regulation. As for the 24 *RCI2* genes in *B. napus*, 20 genes contained abscisic acid response element (ABRE), 9 genes contained defense and stress response element (TC-rich repeats), 17 genes contained anaerobic induced element (ARE), 8 genes contained low-temperature response element (LTR), and 14 genes contained GT1-motif element. These results suggest that the *RCI2* genes in *B. napus* contain many environmental stress elements, which play an important role in the process of stress resistance.

By analyzing the *cis*-acting elements of these 14 orthologous gene pairs (Figure 4), it was found that the numbers of *cis*-acting elements in *B. napus* were more than that of two diploid parent ancestors. Of the eight orthologous gene pairs (*BrRCI2A-a* and *BnARCI2A-a*, *BrRCI2H-a* and *BnCRCI2H-a2*, *BrRCI2H-b* and *BnARCI2H-b2*, *BoRCI2A-a* and *BnCRCI2A-b*, *BoRCI2C* and *BnCRCI2C-b*, *BoRCI2F* and *BnARCI2F-d3*, *BoRCI2H-a* and *BnARCI2H-a1*, *BoRCI2H-b* and *BnCRCI2H-b1*), *B. napus* had the most CAREs types. Of the four orthologous gene pairs (*BrRCI2E* and *BnCRCI2E-a*, BrRCI2F-a and *BnARCI2F-c*, *BoRCI2E* and *BnARCI2E-b*), *B. napus* had the least amount of CAREs types. Two orthologous gene pairs (*BoRCI2A-c* and *BnCRCI2A-c*, *BoRCI2A-a* and *BnCRCI2A-b*) had the same CAREs types. The number of *cis*-acting elements of *RCI2* gene in *B. napus* (585) was greater than the sum of the two diploid progenitors (472), which indicated that the expression regulation in the process of allopolyploidization became more complex.

### 2.9. Analysis of RCI2 Gene Expression

**Figure 7:**
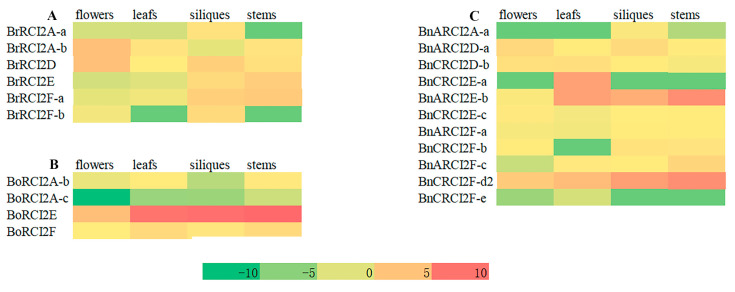
Expression patterns of *RCI2* genes in four tissues (stem, leaf, flower, silique) of *B. rapa* (**A**), *B. oleracea* (**B**) and *B. napus* (**C**).

Based on the RNA-seq data of our research group (Table S4) [39], we investigated the expression patterns of *RCI2* genes in four major tissues (leaves, stems, flowers, and siliques) of allotetraploid *B. napus* and its diploid progenitors (*B. rapa* and *B. oleracea*). For further research, we plotted a heat map to display the gene expression levels in four tissues (Figure 7). The darker the red, the higher the expression level, and the darker the green, the lower the expression level. As can be seen from the heat map (Figure 7), the gene expression of *RCI2* gene family members varied greatly in different tissues, reflecting the diversity of *RCI2* gene functions during growth and development. Among the 42 identified members of the *RCI2* gene family, 21 genes were not detected in the four tissues, indicating that they had spatio-temporal expression specificity. Most of the remaining 21 genes had specific-tissue expression patterns. Specifically, *BrRCI2A-a* was expressed at low levels in stems; *BoRCI2A-c*, *BoRCI2E*, and *BnARCI2E-b* were expressed at low levels in flowers; *BnARCI2A-a* was expressed at low levels in flowers and leaves; *BnARCI2E-b* was expressed at low levels in flowers and leaves; *BnCRCI2E-a* was specifically highly expressed in leaves; and *BnCRCI2F-d2* was specifically highly expressed in siliques and stems.

## 3. Discussion

### 3.1. The BnRCI2 Gene Family Was Amplified in Allotetraploid

Studies have shown that about 80% of gene families in the model plant *A. thaliana* have increased in number during evolution, indicating that gene family amplification has occurred [40]. *B. napus* was obtained from its diploid ancestors *B. rapa* and *B. oleracea* by natural hybridization and chromosome doubling. Thus, the number of *RCI2* gene family members in *B. napus* should be equal to the sum of *B. rapa* and *B. oleracea*. Overall, 15 and 10 *RCI2* genes were identified in *B. rapa* and *B. oleracea*, respectively, and the number of *RCI2* genes in *B. napus* (24) was more than their combined value. Specifically, except for the *BnRCI2As* and *BnRCI2Gs*, the numbers of *BnRCI2* genes were greater than that of the corresponding homologous genes in *B. rapa* and *B. oleracea*, indicating that the *RCI2* gene family of *B. napus* expanded in the allopolyploidization.

Gene replication events are important for the expansion of gene families [41]. However, the main types of replication that lead to the expansion of different families are also not the same. Cannon studied the replication types of 50 gene families in *A. thaliana* and showed that tandem duplication was prominent in some gene families, while segment duplication was more prominent in others [42]. When analyzing the duplication genes, 33 pairs of homologous genes were considered as segment duplicated genes. Two pairs of tandem duplication genes (*BnARCI2F-d3 and BnARCI2E-b*, *BnCRCI2F-d2*, *and BnCRCI2E-a*) were found in the *B. napus* gene family. Overall, segmental duplication is the main driver of the amplification of the *BnRCI2* gene family. In addition, the whole genome replication (WGD) and other single gene replication (DSD, PD, TD, TRD) are also numerous [38]. Unlike diploid ancestors, the TRD mediated by transposon was more than the whole genome replication (WGD) in *B. napus*. This suggests that TRD mediated by transposon may play an important role in the amplification of the *BnRCI2* gene family during allopolyploidization.

### 3.2. The Loss of RCI2 Genes in Diploid Progenitors was Associated with the Process of Diploidization after the WGT Event

In addition to the whole genome duplication (WGD) event that *Brassica* ancestors experienced with Arabidopsis, they also experienced a WGT (whole-genome triplication) event [43]. Therefore, theoretically, each *AtRCI2* gene should correspond to three syntenic genes in diploid *B. rapa* and *B. oleracea*. There should be 24 and 24 syntenic genes in *B. rapa* and *B. oleracea*, respectively. However, only 9 *BrRCI2s* and 9 *BnRCI2s* were identified, which were far less than the number of genes that should be present theoretically, indicating that the large-scale loss of *RCI2* genes in *Brassica* has been lost during the evolution process after the WGT event. One explanation for this is that the subsequent diploidization after a WGT event leads to genome fragmentation, block recombination, and chromosome reduction. One explanation for this is that species undergo genome fractionation, block reshuffling, and chromosome reduction after the WGT event [44,45]. Extensive chromosome rearrangement after WGT mediates the occurrence of neo-polyploid genetic diploidization process, and in the long-term process of natural selection, some fragments with high homology on chromosomes are easily lost, resulting in the loss of some duplicate genes [44,46]. This is a common source of genetic variation and one of the most important evolutionary forces in biology [47]. The gene dose hypothesis has suggested that certain genes undergo dose changes after gene replication and tend to reserve at a relatively low frequency because they may alter the concentration of gene products [48]. There are two main molecular mechanisms that can lead to gene loss in a given genome. Firstly, the loss of a gene can be the result of a mutational event, such as an unequal crossover during meiosis or mobilization of transposable or viral elements that result in the sudden physical removal of the gene from an organism’s genome. Second, the loss of a gene can be the consequence of the slow accumulation of mutations during the pseudogenization after an initial loss-of-function mutation [47].

## 4. Materials and Methods

### 4.1. Materials and Transcriptome Sequencing

The expression data in this study came from the previous sequencing data of our research group [49]. The seeds of the allotetraploid *B. napus* (cv. Darmor) and its diploid progenitors *B. rapa* (cv. Chiifu) and *B. oleracea* (cv. Jinzaosheng) were obtained from the Oil Crops Research Institute, Chinese Academy of Agricultural Sciences, China. These materials were grown under natural conditions in Wuhan, China, and inflorescences were bagged to prevent pollen contamination before blossom. The leaves, stems, flowers, and siliques were then used for Illumina HiSeq X-Ten (San Diego, CA, USA).

### 4.2. Identification of RCI2 Gene Family

Firstly, eight AtRCI2 protein sequences were downloaded from TAIR database (https://www.Arabidopsis.org/, accessed on 2 July 2021) [50], and then submitted to the BRAD database (http://Brassicadb.org/brad/, accessed on 4 July 2021) [51] to BLASTp (*e* value < 1 × 10^5^). The obtained ID were de-duplicated. Then, the integrity of the PMP3 domain was verified by submitting candidate sequences one by one to the following databases: CDD (http://www.ncbi.nlm.nih.gov/Structure/cdd/wrpsb.cgi/, accessed on 5 July 2021) [52], SMART (http://smart.embl-heidelberg.de/, accessed on 5 July 2021) [53], and InterProScan (http://www.ebi.ac.uk/interpro/, accessed on 5 July 2021) [54]. Finally, according to *Brassica* standard nomenclature [55] and referencing the naming of the *AtRCI2* gene families, all *RCI2* genes of *B. rapa*, *B. oleracea* and *B. napus* were renamed.

### 4.3. Chromosomal Mapping, Gene Structures and Gene Duplication Types of the RCI2 Gene Family

The physical location of *RCI2* genes were plotted by the MapChart tool [56]. The gene structures were displayed by GSDS 2.0 (http://gsds.gao-lab.org/, accessed on 10 July 2021) [57]. Gene duplication types were obtained by the Plant Duplicate Gene Database (PlantDGD, http://pdgd.njau.edu.cn:8080, accessed on 12 July 2021) [38].

### 4.4. Conserved Motif and Characteristic Analysis and Subcellular Localization Analysis

Conserved motifs in RCI2 proteins were investigated by online MEME server (http://meme-suite.org/tools/meme, accessed on 13 July 2021) [58]. Moreover, the physico-chemical characteristics of RCI2 proteins in *B. napus* and its two diploid progenitors were calculated by the online ProtParam tool of ExPASy (http://weB.expasy.org/protparam/, accessed on 18 July 2021) [59], including sequence length, molecular weight (MW), theoretical isoelectric point (pI), instability index (II), aliphatic index, and the grand average of hydropathicity (GRAVY). The subcellular localization of RCI2 proteins in *B. napus* and its diploid progenitors was predicted using Cell-PLoc (http://www.csbio.sjtu.edu.cn/bioinf/Cell-PLoc-2/, accessed on 6 November 2021) [60].

### 4.5. Phylogenetic Relationship Analysis

The protein sequences in five species (*B. rapa*, *B. oleracea*, *B. napus*, *A. thaliana*, and *O. sativa*) were aligned by ClustalX [61]. Subsequently, based on Maximum Likelihood (ML), the phylogenetic tree was constructed by MEGA 7.0.26 (bootstrap value: 1000) [62]. Finally, the online Interactive Tree of Life (iTOL, http://itol.embl.de/, accessed on 19 July 2021) was used to decorate this phylogenetic tree [63].

### 4.6. Gene Duplication and Syntenic Analysis

Duplicated *RCI2* genes were identified by BLASTn. If the two *RCI2* gene sequences whose coverage and identity were greater than or equal to 80%, the two *RCI2* genes were regarded as duplicate gene pairs [64]. DnaSP software (version 5.10.01; Geneva, Switzerland) [65] was used to calculate the synonymous (Ks), nonsynonymous (Ka), and Ka/Ks of *RCI2* duplicated gene pairs. The syntenic genes of *RCI2s* in *B. napus* and its two diploid progenitors were searched in the BRAD database, and the syntenic relationship between genes was shown by TBtools [66].

### 4.7. Cis-Elements Analysis

The 2000 bp upstream of the transcription start site (TSS) of the *RCI2* genes were detected to analyze the *cis*-elements in the promoters by using the Plant *cis*-acting Regulatory Element (PlantCARE) server (http://bioinformatics.psb.ugent.be/webtools/plantcare/html/, accessed on 22 July 2021) [67], and then, the result was plotted into a heatmap.

## 5. Conclusions

In this study, 24, 9, and 9 *RCI2* genes were identified in allotetraploid *B. napus*, the A_n_ genome donor *B. rapa*, and the C_n_ genome donor *B. oleracea*, respectively. The structure of *RCI2* genes in the three species was much conserved, and most of them were two exons with an intron. Gene expression patterns and *cis*-acting elements were also analyzed. Whole genome duplication, segmental duplication, and abundant TEs were determined to be the three major impetuses for the expansion of the *RCI2* gene family during the process of polyploidization. Moreover, gene loss events have happened in the *RCI2* gene family in *B. napus* during polyploidization. Additionally, the *RCI2* gene family in *B. napus* was highly conserved at the DNA and protein level, but changed at the expression level during polyploidization. Together, these results can increase our understanding of the evolution of the *RCI2* gene family and provide a reference for future polyploidization analysis.

## Figures and Tables

**Table 1:** Replication types of *RCI2* genes in *B. napus* and its diploid progenitors.

Replication Types	Number		
	*B. rapa*	*B. oleracea*	*B. napus*
WGD	9	5	13
TRD	9	5	14
TD	9	6	10
PD	9	5	7
DSD	8	5	21

## Data Availability

The raw data of RNA-seq reads were deposited in the NCBI database under accession number (SRR7816633-SRR7816668).

## Supplementary Materials

The following are available online at https://www.mdpi.com/article/10.3390/ijms23020614/s1.

## References

[1] Rothfels C.J. (2021). Polyploid phylogenetics. New Phytol..

[2] Doyle J.J., Doyle J.L., Brown A.H. (1999). Origins, colonization, and lineage recombination in a widespread perennial soybean polyploid complex. Proc. Natl. Acad. Sci. USA.

[3] Leitch A.R., Leitch I.J. (2008). Genomic plasticity and the diversity of polyploid plants. Science.

[4] Soltis P.S., Soltis D.E. (2016). Ancient WGD events as drivers of key innovations in angiosperms. Curr. Opin. Plant. Biol..

[5] Adams K.L., Wendel J.F. (2005). Polyploidy and genome evolution in plants. Curr. Opin. Plant. Biol..

[6] Barker M.S., Arrigo N., Baniaga A.E., Li Z., Levin D.A. (2016). On the relative abundance of autopolyploids and allopolyploids. New Phytol..

[7] Cai X., Chang L., Zhang T., Chen H., Zhang L., Lin R., Liang J., Wu J., Freeling M., Wang X. (2021). Impacts of allopolyploidization and structural variation on intraspecific diversification in *Brassica rapa*. Genome Biol..

[8] Jackson S., Chen Z.J. (2010). Genomic and expression plasticity of polyploidy. Curr. Opin. Plant. Biol..

[9] Otto S.P., Whitton J. (2000). Polyploid incidence and evolution. Annu. Rev. Genet..

[10] Doyle J.J., Flagel L.E., Paterson A.H., Rapp R.A., Soltis D.E., Soltis P.S., Wendel J.F. (2008). Evolutionary genetics of genome merger and doubling in plants. Annu. Rev. Genet..

[11] Otto S.P. (2007). The evolutionary consequences of polyploidy. Cell.

[12] Madlung A., Wendel J.F. (2013). Genetic and epigenetic aspects of polyploid evolution in plants. Cytogenet. Genome Res..

[13] Wendel J.F., Lisch D., Hu G., Mason A.S. (2018). The long and short of doubling down: Polyploidy, epigenetics, and the temporal dynamics of genome fractionation. Curr. Opin. Genet. Dev..

[14] Wang X., Zhang Z., Fu T., Hu L., Xu C., Gong L., Wendel J.F., Liu B. (2017). Gene-body CG methylation and divergent expression of duplicate genes in rice. Sci. Rep..

[15] Petit M., Guidat C., Daniel J., Denis E., Montoriol E., Bui Q.T., Lim K.Y., Kovarik A., Leitch A.R., Grandbastien M.A. (2010). Mobilization of retrotransposons in synthetic allotetraploid tobacco. New Phytol..

[16] Kashkush K., Feldman M., Levy A.A. (2002). Gene loss, silencing and activation in a newly synthesized wheat allotetraploid. Genetics.

[17] Liu S., Yang Y., Wei F., Duan J., Braynen J., Tian B., Cao G., Shi G., Yuan J. (2017). Autopolyploidy leads to rapid genomic changes in *Arabidopsis thaliana*. Theory Biosci..

[18] Zhao L., Han L., Xiao C., Lin X., Xu C., Yang C. (2018). Rapid and pervasive development and tissue-specific homeolog expression partitioning in newly formed inter-subspecific rice segmental allotetraploids. BMC Genom..

[19] Kim Y.O., Kim H.S., Lim H.G., Jang H., Kim E., Ahn S.J. (2021). Functional characterization of salt-stress induced rare cold inducible gene from *Camelina sativa* (*CsRCI2D*). J. Plant Biol..

[20] Mitsuya S., Taniguchi M., Miyake H., Takabe T. (2005). Disruption of RCI2A leads to over-accumulation of Na^+^ and increased salt sensitivity in *Arabidopsis thaliana* plants. Planta.

[21] Fu J., Zhang D.F., Liu Y.H., Ying S., Shi Y.S., Song Y.C., Li Y., Wang T.Y. (2012). Isolation and characterization of maize PMP3 genes involved in salt stress tolerance. PLoS ONE.

[22] Kwok A.C.M., Zhang F., Ma Z., Chan W.S., Yu V.C., Tsang J.S.H., Wong J.T.Y. (2020). Functional responses between PMP3 small membrane proteins and membrane potential. Environ. Microbiol..

[23] Ben Romdhane W., Ben-Saad R., Meynard D., Verdeil J.L., Azaza J., Zouari N., Fki L., Guiderdoni E., Al-Doss A., Hassairi A. (2017). Ectopic expression of *Aeluropus littoralis* plasma membrane protein gene *AlTMP1* confers abiotic stress tolerance in transgenic tobacco by improving water status and cation homeostasis. Int. J. Mol. Sci..

[24] Navarre C., Goffeau A. (2000). Membrane hyperpolarization and salt sensitivity induced by deletion of PMP3, a highly conserved small protein of yeast plasma membrane. EMBO J..

[25] Zhao Y., Tong H., Cai R., Peng X., Li X., Gan D., Zhu S. (2014). Identification and characterization of the *RCI2* gene family in maize (*Zea mays*). J. Genet..

[26] Nylander M., Heino P., Helenius E., Palva E.T., Ronne H., Welin B.V. (2001). The low-temperature- and salt-induced *RCI2A* gene of Arabidopsis complements the sodium sensitivity caused by a deletion of the homologous yeast gene *SNA1*. Plant. Mol. Biol..

[27] Ben-Romdhane W., Ben-Saad R., Meynard D., Zouari N., Mahjoub A., Fki L., Guiderdoni E., Al-Doss A., Hassairi A. (2018). Overexpression of *AlTMP2* gene from the halophyte grass *Aeluropus littoralis* in transgenic tobacco enhances tolerance to different abiotic stresses by improving membrane stability and deregulating some stress-related genes. Protoplasma.

[28] Nagaharu U., Nagaharu N. (1935). Genome analysis in *Brassica* with special reference to the experimental formation of *B. napus* and peculiar mode of fertilization. Jpn. J. Bot..

[29] Medina J., Ballesteros M.L., Salinas J. (2007). Phylogenetic and functional analysis of Arabidopsis *RCI2* genes. J. Exp. Bot..

[30] Zhou Y., Ge L., Li G., He P., Yang Y., Liu S. (2020). In silico identification and expression analysis of Rare Cold Inducible 2 (*RCI2*) gene family in cucumber. J. Plant Biochem. Biotechnol..

[31] Brunetti S.C., Arseneault M.K.M., Gulick P.J. (2018). Characterization of the *Esi3*/*RCI2*/*PMP3* gene family in the Triticeae. BMC Genom..

[32] Morsy M.R., Almutairi A.M., Gibbons J., Yun S.J., de Los Reyes B.G. (2005). The *OsLti6* genes encoding low-molecular-weight membrane proteins are differentially expressed in rice cultivars with contrasting sensitivity to low temperature. Gene.

[33] Kim S.H., Kim J.Y., Kim S.J., An K.S., An G., Kim S.R. (2007). Isolation of cold stress-responsive genes in the reproductive organs, and characterization of the *OsLti6b* gene from rice (*Oryza sativa* L.). Plant Cell Rep..

[34] Kim H.S., Park W., Lee H.S., Shin J.H., Ahn S.J. (2021). Subcellular journey of rare cold inducible 2 protein in plant under stressful condition. Front. Plant Sci..

[35] Rocha P.S. (2016). Plant abiotic stress-related *RCI2/PMP3s*: Multigenes for multiple roles. Planta.

[36] Yeshvekar R.K., Nitnavare R.B., Chakradhar T., Bhatnagar-Mathur P., Reddy M.K., Reddy P.S. (2017). Molecular characterization and expression analysis of pearl millet plasma membrane proteolipid 3 (*Pmp3*) genes in response to abiotic stress conditions. Plant Gene.

[37] Cheng F., Wu J., Fang L., Wang X. (2012). Syntenic gene analysis between *Brassica rapa* and other Brassicaceae species. Front. Plant Sci..

[38] Qiao X., Li Q., Yin H., Qi K., Li L., Wang R., Zhang S., Paterson A.H. (2019). Gene duplication and evolution in recurring polyploidization-diploidization cycles in plants. Genome Biol..

[39] Li M., Wang R., Liu Z., Wu X., Wang J. (2019). Genome-wide identification and analysis of the WUSCHEL-related homeobox (WOX) gene family in allotetraploid *Brassica napus* reveals changes in *WOX* genes during polyploidization. BMC Genom..

[40] Lespinet O., Wolf Y.I., Koonin E.V., Aravind L. (2002). The role of lineage-specific gene family expansion in the evolution of eukaryotes. Genome Res..

[41] Liu S., Liu Y., Yang X., Tong C., Edwards D., Parkin I.A., Zhao M., Ma J., Yu J., Huang S. (2014). The Brassica oleracea genome reveals the asymmetrical evolution of polyploid genomes. Nat. Commun..

[42] Cannon S.B., Mitra A., Baumgarten A., Young N.D., May G. (2004). The roles of segmental and tandem gene duplication in the evolution of large gene families in *Arabidopsis thaliana*. BMC Plant Biol..

[43] Lysak M.A., Koch M.A., Pecinka A., Schubert I. (2005). Chromosome triplication found across the tribe Brassiceae. Genome Res..

[44] Cheng F., Wu J., Wang X. (2014). Genome triplication drove the diversification of *Brassica* plants. Hortic. Res..

[45] Wang L., Jia G., Jiang X., Cao S., Chen Z.J., Song Q. (2021). Altered chromatin architecture and gene expression during polyploidization and domestication of soybean. Plant Cell.

[46] Paterson A.H., Bowers J.E., Chapman B.A. (2004). Ancient polyploidization predating divergence of the cereals, and its consequences for comparative genomics. Proc. Natl. Acad. Sci. USA.

[47] Albalat R., Cañestro C. (2016). Evolution by gene loss. Nat. Rev. Genet..

[48] Freeling M. (2008). The evolutionary position of subfunctionalization, downgraded. Genome Dyn..

[49] Li M., Wang R., Wu X., Wang J. (2020). Homoeolog expression bias and expression level dominance (ELD) in four tissues of natural allotetraploid *Brassica napus*. BMC Genom..

[50] Poole R.L. (2007). The TAIR database. Methods Mol. Biol..

[51] Wang X., Wu J., Liang J., Cheng F., Wang X. (2015). *Brassica* database (BRAD) version 2.0: Integrating and mining Brassicaceae species genomic resources. Database.

[52] Marchler-Bauer A., Bo Y., Han L., He J., Lanczycki C.J., Lu S., Chitsaz F., Derbyshire M.K., Geer R.C., Gonzales N.R. (2017). CDD/SPARCLE: Functional classification of proteins via subfamily domain architectures. Nucleic Acids Res..

[53] Schultz J., Copley R.R., Doerks T., Ponting C.P., Bork P. (2000). SMART: A web-based tool for the study of genetically mobile domains. Nucleic Acids Res..

[54] Zdobnov E.M., Apweiler R. (2001). InterProScan-an integration platform for the signature-recognition methods in InterPro. Bioinformatics.

[55] Ostergaard L., King G.J. (2008). Standardized gene nomenclature for the *Brassica* genus. Plant Methods.

[56] Voorrips R.E. (2002). MapChart: Software for the graphical presentation of linkage maps and QTLs. J. Hered..

[57] Hu B., Jin J., Guo A.Y., Zhang H., Luo J., Gao G. (2015). GSDS 2.0: An upgraded gene feature visualization server. Bioinformatics.

[58] Bailey T.L., Boden M., Buske F.A., Frith M., Grant C.E., Clementi L., Ren J., Li W.W., Noble W.S. (2009). MEME SUITE: Tools for motif discovery and searching. Nucleic Acids Res..

[59] Chou K.C., Shen H.B. (2008). Cell-PLoc: A package of Web servers for predicting subcellular localization of proteins in various organisms. Nat. Protoc..

[60] Waterhouse A., Bertoni M., Bienert S., Studer G., Tauriello G., Gumienny R., Heer F.T., de Beer T.A.P., Rempfer C., Bordoli L. (2018). SWISS-MODEL: Homology modelling of protein structures and complexes. Nucleic Acids Res..

[61] Thompson J.D., Gibson T.J., Higgins D.G. (2002). Multiple sequence alignment using ClustalW and ClustalX. Curr. Protoc. Bioinform..

[62] Kumar S., Stecher G., Tamura K. (2016). MEGA7: Molecular evolutionary genetics analysis version 7.0 for bigger datasets. Mol. Biol. Evol..

[63] Letunic I., Bork P. (2021). Interactive Tree of Life (iTOL) v5: An online tool for phylogenetic tree display and annotation. Nucleic Acids Res..

[64] Kong X., Lv W., Jiang S., Zhang D., Cai G., Pan J., Li D. (2013). Genome-wide identification and expression analysis of calcium-dependent protein kinase in maize. BMC Genom..

[65] Librado P., Rozas J. (2009). DnaSP v5: A software for comprehensive analysis of DNA polymorphism data. Bioinformatics.

[66] Chen C., Chen H., Zhang Y., Thomas H.R., Frank M.H., He Y., Xia R. (2020). TBtools: An integrative toolkit developed for interactive analyses of big biological data. Mol. Plant.

[67] Lescot M., Déhais P., Thijs G., Marchal K., Moreau Y., Van de Peer Y., Rouzé P., Rombauts S. (2002). PlantCARE, a database of plant *cis*-acting regulatory elements and a portal to tools for in silico analysis of promoter sequences. Nucleic Acids Res..

